# Application of Microbial Bioenzymes in Soil Stabilization

**DOI:** 10.1155/2020/1725482

**Published:** 2020-07-27

**Authors:** Eshetu Mekonnen, Ameha Kebede, Tekle Tafesse, Mesfin Tafesse

**Affiliations:** ^1^Dire Dawa University, College of Natural and Computational Sciences, Department of Biology, Dire Dawa, Ethiopia; ^2^Haramaya University, College of Natural and Computational Sciences, School of Biology and Biotechnology, Haramaya, Ethiopia; ^3^Addis Ababa Science and Technology University, Department of Biotechnology, Addis Ababa, Ethiopia

## Abstract

Soil stabilization is a mechanical or chemical alteration of one or more soil properties to create an improved soil material possessing the desired engineering properties. The aim of this article was to review bioenzyme-based soil stabilization techniques with an emphasis on bioenzymes production, mechanism of soil stabilization and future challenges, and opportunities of the sector. Soils are stabilized to increase strength and durability or to prevent erosion and dust generation. Cost-effective soil stabilization technology has been a fundamental part of any construction and is very important for economic growth in any country. In some cases, construction has been challenged due to the high cost of soil stabilization processes. Besides, methods of stabilizations using common stabilizing agents are getting costly. Currently, there is a growing interest to identify new and green technology to improve construction techniques and to expand the road network. Therefore, the search for new materials and improved techniques to process the local materials has received an increased focus. For developing countries, bioenzymes are now creating an opportunity to improve soil stability with tremendous effectiveness in the overall process of soil stabilization. In the world, bioenzymes have been used in different projects for several years and are generally proprietary products, often of patented formulation that needs intensive field tests. Currently, the use and production of bioenzymes is becoming the most promising key for the advancement of a country by saving time, energy, and finance. It also reduces environmental pollution due to carbon emission by the conventional stabilizers. Thus, a better understanding of this emerging technology is of utmost importance to exploit any improvement it can offer to soil stability. With little research and practice, it is possible to produce soil stabilizing bioenzymes using local raw materials. Due to this, production of low cost, easily and widely applicable, and environmentally friendly enzymatic formulations from locally available raw materials should be the interest of research and academic institutes of any country.

## 1. Introduction

Soil stabilization is the permanent physical and chemical alteration of soils to enhance their physical properties [1]. In its broadest senses, it includes compaction, preconsolidation, drainage, and many such processes. However, the term stabilization is generally restricted to the process which alters the soil material itself for improvement of its properties [2]. It is the collective term for any physical, chemical, or biological method, or combination of such methods employed to improve certain properties of natural soil to make it serve for intended engineering purposes [3]. Improvements include increasing the dry unit weight, bearing capabilities, volume changes, the performance of *in situ* subsoils, sands, and other waste materials in order to strengthen road surfaces, and other geotechnical applications [4]. It is required when the soil available for construction is not suitable for the intended purpose and mainly aimed at increasing resistance to softening by water through bonding the soil particles together, water proofing the particles, or combination of the two [5].

The concept of stabilization is 5,000 years old [4] and has been considered as old as construction has existed [6]. Ancient Chinese, Romans, and Icas buildings and road ways which existed till today utilized different techniques of soil stabilization [7]. Stabilized Earth roads were used in ancient Egypt and Mesopotamia and the Greeks and Romans used lime as a stabilizer. Thus, building material dates back 5,000 years when lime and clay were mixed and compacted to form bricks used in the construction of the pyramids and about 2,000 years ago when the Romans used lime to improve the quality of their roads [8]. The first soil stabilization tests were performed in the United States in 1904 [9].

The modern era of soil stabilization began during the 1960s and 1970s when general shortages of aggregates and fuel resources forced engineers to consider alternatives to the conventional techniques of replacing poor soils at building sites with shipped-in aggregates that possessed more favorable engineering characteristics [10]. The modern stabilization techniques are aimed at assuring adequate subgrade stability, especially for weaker soils.

Soil stabilization is generally costly and requires huge investments. In some cases, construction has been hindered due to the high cost of soil stabilization techniques and depletion of stabilizing materials [4]. The development of cost-effective materials and processes has been a crucial part of any construction for years. Hence, cost-effective road construction techniques are vital for economical growth in any country. As a result, there is an urgent need to identify new and cost-effective materials to improve construction techniques and expand road networks. Recently, the search for new materials and improved techniques for processing the local materials has received an increased interest. In the past decades, a number of organic and inorganic chemical additives and different standard soil stabilizers like hydrated lime, Portland cement, and bitumen have been developed worldwide [11–14]. However, more attention has recently been given to the use of bioenzymes as soil stabilizers.

## 2. Microbial Enzymes (Bioenzymes) as a Soil Stabilizer

Enzymes are the catalysts of biological systems that not only control the rate of reactions but also can lower the activation energy for the formation of one product from another by favoring certain geometries in the transition state [15]. Bioenzymes are protein molecules that catalyze chemical reactions in the soil to form a cementing bond that stabilizes the soil structure and reduces the soil's affinity for water [16]. The idea of using enzyme stabilization for soil pavement was developed from the application of enzyme products used to treat soil in order to improve horticultural applications [17]. A modification to the process produced a material suitable for stabilization of poor ground for road traffic.

Bioenzymes work on a variety of soils as long as the minimum amount of clay particles is present [18, 19]. According to Khan and Taha [19], enzymes may work suitably for soils containing 12–24% clay fraction with a plasticity index between 8 and 35. When applied at low application rates to the surface of the unbound road surface, enzymatic emulsions perform well for dust suppression [20]. At higher application rates, enzymatic emulsions can be used to stabilize unpaved and paved roads, paths and shoulders, access roads, unpaved and paved parking lots, orchards and crop roads, mining haul roads, access roads, parking areas, airfields, minor rural roads, property driveways, and where you need to improve the engineering properties of road bed materials [21]. When applied and compacted properly, the treated soils can be stabilized to form a dense, firm-to-hard, water-resistant bound layer that can be used as a road surfacing.

## 3. Comparison between Bioenzymes and Traditional Stabilizers

Traditional stabilizers such as cement and lime are relatively expensive and in some areas the cost would be up to three times the cost of bioenzymes and become even more so when they have to be transported long distances to low-volume road construction sites, because they are bulky [22]. On the other hand, bioenzymes are usually sold as concentrated liquids, diluted with water at the construction site and then either spread on the soil before compaction or pressure injected to treat deeper soil layers [23]. Due to this fact, it is possible to transport with relatively reduced price. Because of the lower transportation costs, concentrated bioenzymes can be an attractive alternative for stabilization projects. As a consequence, unlike the traditional soil stabilization techniques, bioenzymes are the cheapest, nontoxic, environmentally friendly, and organic technology.

As a consequence, recently more attention has been given to the use of bioenzymes as soil stabilizers. This is due to the expansion in manufacturing capacity, low cost, and relatively wide applicability of the enzymes as compared to standard stabilizers requiring large amounts of stabilizers to stabilize soils which in turn increases manufacturing cost.

## 4. Clay-Water Interaction

The major problems for soil stability during any construction is the nature of the clay constituting the soil mass. Some clays exhibit significant volume changes due to the variation of water content in the mass of the soil [24, 25], in response to climatic conditions and the action of vegetation [26, 27], and such soils are called expansive soils [28, 29]. Expansive soil is a term which is applied to the soils that expand in the presence of water and shrink when they dry out [24, 30–33]. They are clayey soils with a high specific surface area and cation exchange capacity that usually have a predominant clay mineral and the soil is of the swelling lattice type, montmorillonite [31, 34, 35], and usually contain more than 30% clay to a minimum depth of 50 cm [36].

**Figure 1:**
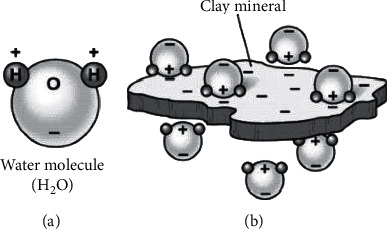
Clay particle and surface charge display [31].

Clayey soils have a high affinity for water because of their small particle size and high surface activity [31]. Thus, the particles are almost always hydrated, that is, surrounded by layers of water molecules adsorbed onto the clay particles. This affinity for water can be attributed to hydrogen bonding (oxygen or hydroxyl molecules attract the hydrogen of water), Van der Waals attractions, and charged surface-dipole attractions (Figure 1) [37]. It is this water layer that affects all soil properties including plasticity, compaction, strength, and water movement in the soil [38]. Among these different types of bonding, the hydrogen bonding is the strongest and is considered to be the primary reason behind the swelling of expansive soils due to water absorption [39]. Due to this, montmorillonite clays suffer volume changes due to moisture content changes which results in swelling and shrinkage [10]. This phenomenon is influenced by many clay properties including specific surface area, cation exchange capacity, organic matter content, and availability of soil stabilizing agents. Soil stabilizers bond soil minerals together and lead to suppression of swelling by increasing strength to the soil material [40].

**Figure 2:**
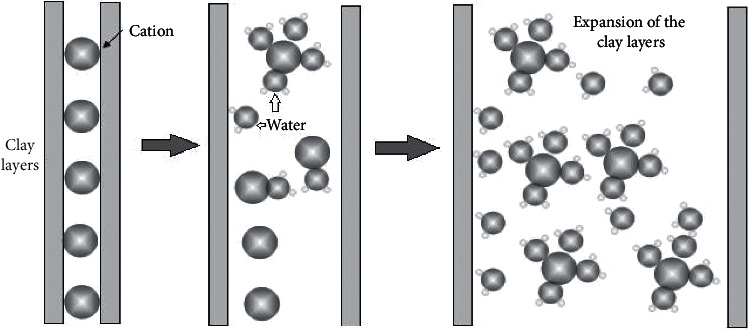
Clay-water interaction.

The clay particles hold a high concentration of cations to balance the negative surface charges attributed to the presence of broken bonds and isomorphous substitution. These cations are termed as “adsorbed cations” and are strongly held by the negatively charged clay particles [31]. The cations tend to diffuse away from the clay surface in order to balance the low cation concentration within the absorbed water. However, this kind of diffusion is offset by the electrostatic attraction between the positively charged cations to the negatively charged clay surface, which is more dominant close to the clay particles. The negatively charged clay surface, along with the strongly held cations (close to the clay particle) and the relatively loosely held diffused cations (further away from the clay particle), form the diffuse double layer [39]. This diffuse double layer governs the clay-water interaction and affects the engineering properties of clay, including swelling and plasticity [41] (Figure 2).

## 5. Mechanism of Bioenzyme Soil Stabilization

Unlike traditional stabilizers, the attempts done to define the stabilization mechanisms of nontraditional stabilizers including bioenzymes have been limited [16]. Several articles were published on laboratory and field experimentations with bioenzymes. Quite a lot of such publications were focused on performance evaluation instead of mechanism identification [17, 22, 42, 43]. Thus, there is relatively little literature concerning stabilization mechanisms of bioenzymes in soil stabilization. Two mechanisms of bioenzyme soil stabilization were proposed by researchers [44].

The first proposed mechanism of stabilization explained that the enzymes that are present in treated soil are adsorbed by the clay lattice, and in turn cations are released as an exchange, a process similar to cation exchange. This leads to a reduction in the thickness of diffuse double layer of the clay [45], Scholen, 1995 [16]. The other widely accepted hypothesis of bioenzyme soil stabilization mechanism was proposed by Scholen [15]. Scholen proposed that when bioenzyme formulations are mixed with soil, enzymes combine with big organic molecules in the soil solution to generate a reactant mediator. The large organic molecules have large flat structures that approach the size of small clay particles which can blanket the clay minerals, neutralizing the negative charge and reducing the clay's affinity for moisture. As a result, this produces a covering effect, which blocks further absorption of water and loss in density. This reaction regenerates the enzymes again and helps the process to continue repeatedly.

Several researchers showed the formation of stable clay lattice structure and a reduced affinity for moisture after treatment with various bioenzymatic formulations. Rauch et al. [46], through different chemical and physical tests, endorsed the hypothesis proposed by Scholen [15] stating that enzymes unite with the large organic molecules and adhere to clay surfaces, thus jamming potential cation exchange sites and preventing absorption of moisture and subsequent swelling. In addition, in their separate studies, Santoni et al. [47], Tingle et al. [16], Tingle et al. [16], and Tingle and Santoni [48] reported a series of laboratory tests with various bioenzymatic stabilizers evaluating the performance effects in terms of increased strength improvement in both granular and fine-grained subgrade materials. However, these experiments only categorized the proposed stabilization mechanisms as either a mechanical bonding or a chemical reaction mechanism with no details of the proposed physicochemical changes.

However, Lindenbaum [49], Rauch et al. [50], Stan and Ciobanu [43], and John et al. [51] suggested that soil suitable for bioenzyme stabilization should have chemical substances like clay minerals that may react with other chemicals. They indicated that enzymes are appropriate only for use with clay materials that have an affinity for water, particularly high-plasticity clays with some organic content. Thus, materials such as silts and granular soils would not possess a significant affinity for water and would be unsuitable for stabilization with enzyme products [16]. In addition, literatures suggested that the use of enzymes will also be critically dependent on the environmental conditions and may take considerable time to occur [16, 52]. Rauch et al. [50], through different chemical and physical tests, endorsed the hypothesis proposed by Scholen [15] stating that enzymes unite with the large organic molecules and adhere to clay surfaces, thus jamming potential cation exchange sites and preventing absorption of moisture and subsequent swelling.

Lindenbaum [49] in his patent publication also explained a mechanism that bioenzyme during soil stabilization breaks down the electric double layer between the clay and static (adsorbed) water. By this, the clay particles lose its inherent charge and loose the adhered static water layer. In this mechanism, the clay particles segregate and are so fixed crystallographically that it prevents any further volume changes on exposure to water. He also added that organic cations generated by the growth of vegetation and microorganisms will have the capability to exchange position with other ions attracted to the clay platelet in the soil. In contrast to metal cations, the organic cations have large flat structures that approach the size of small clay particles. These organic cations can blanket the clay particle and effectively neutralize its negative charge in a short distance, thus greatly reducing the double layer thickness [16]. Lindenbaum [49] also explained that lowering the dipole moment of the water molecule by the enzyme results in dissociation into a hydroxyl (−) and a hydrogen (+) ion. This will clear the water molecules out of the intermolecular spaces of clay minerals.

## 6. Microbial-Based Bioenzyme Production

Several commercial bioenzymes formulations are available in the market worldwide and have been used for road construction projects [19]. Even though they are produced in large scales in different countries, the formulations of the products are not made public due to commercial proprietary issues. In addition, no published literature is available that explains the detailed procedures and the required recipe in the manufacturing process. The only available publication is a patent publication by Lindenbaum [49]. Lindenbaum reported that the enzyme composition used for soil stabilization included an enzyme expressed by microorganisms produced via fermentation. He also noted that such microorganisms include bacteria and fungi and urolytic groups are selected. According to him, a crop plant biomass could be used as substrate for fermentation. Cuisinier and Masrouri [53] mentioned that enzymatic formulations for soil stabilizations are derived from the fermentation of sugar molasses, a waste of the sugar industry. Khan and Taha [19] reported that soil stabilizing bioenzymes are organic and nontoxic formulations which are generally extracted by the fermentation of vegetables and sugar canes and thus are degradable; that is, they easily break down and dissolve with time.

Enzymes are naturally found in all organisms including unicellular microbes. Each single strain of microorganism can produce a large number of enzymes, hydrolyzing, oxidizing, or reducing and metabolic in nature [54]. DeJong et al. [55] reported that subsurface microbes can promote several biogeochemical reaction networks such as urea hydrolysis, nitrate reduction, sulfate-reduction, and iron reduction. However, the absolute and relative amounts of the various individual enzymes produced vary between species and even between strains of the same species. Hence, it is customary to select strains for the commercial production of specific enzymes which have the capacity for producing highest amounts of the particular enzymes desired. Fujita et al. [56] reported that urolytic bacteria are commonly found in the subsurface and are known to undergo urea hydrolysis to induce calcium carbonate precipitation.

A number of aerobic bacteria species have the capability to breakdown urea in the soil under aerobic conditions, namely, *Proteus, Morganella, Serratia, Pseudomonas, Clostridium, Fusobacterium, Ureaplasma, Providencia,* and *Sarcina* [57]. DeJong et al. [58] also identified urease producing bacteria are from the genera *Bacillus, Sporosarcina, Sporolactobacillus, Clostridium,* and *Desulfotomaculum*. Alizadeh et al. [59] reported the identification of urolytic bacteria from the genera *Citrobacter, Enterobacter, Pseudomonas, Serratia,* and *Yersinia*. Lindenbaum [49] noted that fermentation can be done through incubation of these microorganisms together with a crop plant biomass under conditions suitable for growth of the microorganisms. The selected microorganisms may then secrete a plurality of exoenzymes in a harvestable amount and concentration.

## 7. Commercial Bioenzyme Products

The idea of using enzyme for stabilization in pavement construction was developed from the application of enzyme products used to treat soil in order to improve horticultural applications [52]. The other concept was also believed to be derived from the stabilization technique demonstrated by termites and ants. It was reported that ants and termites saliva is full of enzymes and is used to build soil structures, which are rock hard and meters high. These structures are known to stand firm despite heavy tropical rain seasons [45]. This basic concept has been modified and used to produce a several commercial products which are aimed at stabilization of problematic soils mainly in road construction.

Currently, there are quite a lot of commercial enzymatic products available in the market (Table 1). These formulations have been used in different projects for several years. They are generally proprietary products, often of patented formulation, that do not meet any relevant formal standard specification (e.g., ASTM or AASHTO) and are not covered in any country road authority design guidelines or specifications documentation [62]. These products have been in development since the 1960s, with many research papers and projects having been written on the subject. Despite the large quantity of information available, broad acceptance of these stabilization products has not occurred. Rauch et al. [46] identified several reasons for this, but those reasons are predominantly from the perspective of road agencies and do not include barriers that may be present within the agencies, as experienced from the perspective of product suppliers. These fermentation formulated products are reported as nontoxic and environmentally harmless [16]. Commonly used commercial bioenzyme products for soil stabilization and maintenance are listed below.

## 8. Application of Commercial Bioenzymes for Road Construction in Africa

Some commercial bioenzyme formulations are now being introduced into African markets and are under experimental tests in some countries. Intensive studies on permazyme were conducted in South Africa and Uganda, while experimental study is currently being conducted in Ethiopia at Addis Ababa Science and Technology University in collaboration with Ethiopian Roads Authority. Zyme-Tech and EcoRoads were also introduced and tested in some countries including Ethiopia. The information regarding the application of other soil stabilizing bioenzymes other than permazyme is rare and not well-organized in Africa.

## 9. Future Prospects of Soil Stabilizing Enzymes

Bioenzymatic soil stabilization is now gaining tremendous ground and has a universal approval with institutions like WHO and UNESCO. The main feature of bioenzyme soil stabilization is that it uses no foreign stabilizing material. This aspect opens a great opportunity to improve soil stabilization process with an effective cost reduction in the overall process. Due to its huge economic impact and nontoxicity on the environment, bioenzyme holds the most promising key for developing countries. Bioenzyme technology is advantageous for any country in that it saves stupendous time, energy, and finances. A better understanding of this emerging technology is of utmost importance to exploit any improvement it can offer for betterment of our wellbeing and surroundings. Finally, it is believed that relevant inventions must be identified and commercialized to suit the needs of soil stabilization.

## 10. Conclusions

The content of this paper highlights the function of microbial-based bioenzymes in soil stabilization with an emphasis on expansive soils. The paper mainly tried to discuss bioenzymes as a soil stabilizer, the mechanisms of bioenzyme soil stabilization, and bioenzyme production technology. In general, the following was concluded.

- Soil stabilization is a very crucial procedure in any contraction projects and needs complex technology which produces a stable base that can carry traffic loads.
- The higher price of the chemical and mechanical stabilization techniques has created the need for safe, cheap, and easily produced soil stabilization techniques. Due to this, local production of bioenzymes is the best choice where cost-effective technologies are the primary interest of the economy.
- Enzymes as soil stabilizers have been used to improve the strength of subgrades due to low cost and relatively wide applicability compared to standard stabilizers.
- The use of enzymes as stabilizer has not been subjected to any technical development and is presently carried out using empirical guidelines based on previous experience.
- Currently, several commercial enzymes are available in the market. However, their production procedures and microbes used for the fermentation process are either patent protected or are not easily available to access.
- Because of the variability in nature of soils, it becomes an important priority to study and determine the effects of enzymes on the strength of different soils prior to being used. The production of low cost, easily and widely applicable, and environmentally friendly enzymatic formulations from locally available raw materials should be the interest of research and academic institutes of any country.

## Figures and Tables

**Table 1:** List of commercially available soil stabilizer bioenzyme formulations.

Product name	Supplier	Reference
ClayPack/DuraPack	Soil Bond International Texas	Rauch et al. [46]
Corchem 5510	CORCHEM® corporation, Texas	http://www.corchem.com
Earthzyme	Cypher Environmental Ltd, Winnipeg, Manitoba, Canada	http://www.cypherenvironmental.com
ECOroads	Terrafusion International, inc.	https://www.ecoroads.company/
EMC2	Soil Stabilization Products Co. Merced, California	Rauch et al. [46]
Fujibeton	Japan	Chander [60]
Paczyme	Rainstorm Dust Control (Pty) Ltd.	Ian Steeves [61]
PCS-320	Alpha Omega Enterprises	Rauch et al. [46]
Permazyme	Pacific Enzymes Inc.	Rauch et al. [46]
Renolith	Renolith Technology Corporation, Thailand	http://www.renolitech.com/contactus.asp
Terrazyme	Concord (USA) Ltd., Columbia	—
UBIX 010	Enzymes Plus , Anderson Affiliates Inc.	—
Zyme-Tech	Zyme Technologies, Iceland	info@zymetech.com
Endurazyme 388	World Enzymes Australia, A Division of Mitebridge Pty Ltd.	http://www.ozemail.com.au/∼quadron/roadfrme.htm

## Data Availability

No data were used to support this study.
